# Longitudinal Assessment of Transorbital Sonography, Visual Acuity, and Biomarkers for Inflammation and Axonal Injury in Optic Neuritis

**DOI:** 10.1155/2017/5434310

**Published:** 2017-09-11

**Authors:** Piergiorgio Lochner, Roberto Cantello, Klaus Fassbender, Martin Lesmeister, Raffaele Nardone, Antonio Siniscalchi, Nausicaa Clemente, Andrea Naldi, Lorenzo Coppo, Francesco Brigo, Cristoforo Comi

**Affiliations:** ^1^Department of Neurology, Saarland University Medical Center, Homburg, Germany; ^2^Department of Translational Medicine, Section of Neurology, University of Piemonte Orientale, Novara, Italy; ^3^Division of Neurology, “Franz Tappeiner” Hospital, Merano, Italy; ^4^Department of Neurology, Christian Doppler Medical Centre, Paracelsus Medical University Salzburg, Salzburg, Austria; ^5^Department of Neurology, “Annunziata” Hospital, Cosenza, Italy; ^6^Department of Neuroscience, Biomedicine and Movement Sciences, University of Verona, Verona, Italy

## Abstract

**Background and Objective:**

To investigate the relationship between optic nerve sheath diameter, optic nerve diameter, visual acuity and osteopontin, and neurofilament heavy chain in patients with acute optic neuritis.

**Patients and Methods:**

Sonographic and visual acuity assessment and biomarker measurements were executed in 23 patients with unilateral optic neuritis and in 19 sex- and age-matched healthy controls.

**Results:**

ONSD was thicker on the affected side at symptom onset (median 6.3 mm; interquartile range 6.0–6.5) than after 12 months (5.3 mm; 4.9–5.6; *p* < 0.001) or than in controls (5.2 mm; 4.8–5.5; *p* < 0.001). OND was significantly increased in the affected side (3.4 mm; 2.9–3.8) compared to healthy controls (2.7 mm; 2.5–2.9; *p* < 0.001) and was thicker at baseline than after 12 months (2.8 mm; 2.7–3.0; *p* < 0.01). Visual acuity improved significantly after 12 months (1.00; 0.90–1.00) compared to onset of symptoms (0.80; 0.40–1.00; *p* < 0.001). OPN levels were significantly higher in patients at presentation (median 6.44 ng/ml; 2.05–10.06) compared to healthy controls (3.21 ng/ml, 1.34–4.34; *p* < 0.03). Concentrations of NfH were significantly higher in patients than in controls.

**Conclusion:**

ONSD and OND are increased in the affected eye. OPN and NfH are elevated in patients, confirming the presence of any underlying inflammation and axonal injury.

## 1. Introduction

Optic neuritis can be easily investigated because of its well-defined onset and accessibility to different diagnostic methods [1]. The most common pathophysiological mechanism of optic neuritis is inflammatory demyelination of the optic nerve. However, axonal degeneration and inflammation coexist in these patients and are associated with visual function impairment [2, 3].

Although optic neuritis is a clinical diagnosis, investigations such as retinal optical coherence tomography (OCT) or MRI may be proven useful in supporting the diagnosis. These techniques have provided fascinating insights into the pathophysiology of optic neuritis in its various forms [4, 5]. Transorbital sonography is a sensitive, highly accessible, user-friendly, and reliable technique for detecting optic nerve diameter (OND) and optic nerve sheath diameter (ONSD) [6] and is also able to show a significant thickening of OND and ONSD in the affected side, which is probably due to inflammation with subsequently increased perineural subarachnoid fluid [7].

Transorbital sonography and OCT provide different and complementary information on the pathophysiology of optic neuritis. Compared to OCT, nerve ultrasonography has an inferior resolution but can provide a better depiction of the nerve and the orbita, because OCT is limited to surface analysis [7].

In a recent systematic review assessing the role of optic ultrasonography in the diagnosis of acute optic neuritis, this technique was able to detect an increase of the optic nerve and sheath diameters in about 80% of affected patients [8]. This finding is consistent with the development of vasogenic edema and the presence of the early optic nerve lesion, recognizable by the gadolinium leakage often shown in MRI or the lesions identified with particular sequences of MRI such as FLAIR sequences [5, 9].

Conversely, OCT accurately documents changes in the thickness of retinal layers such as swelling in case of acute optic neuritis with optic disc swelling; this might be proven further useful, although only one-third of patients with acute optic neuritis show papillitis [4, 8].

Osteopontin (OPN), a proinflammatory cytokine expressed in several tissues and pathological conditions [10, 11], was shown to be increased in the cerebrospinal fluid of patients with optic neuritis [12]. In multiple sclerosis (MS), OPN levels correlate with disease severity and relapse rate [13]. On the other hand, blood levels of neurofilament heavy chain (NfH), a biomarker for neurodegeneration, are increased in patients with acute optic neuritis and inversely correlated with visual loss [14]. Simultaneous assessment of visual acuity, transorbital sonography, and biomarkers may provide an opportunity to further explore inflammation and neurodegeneration in optic neuritis and was therefore the aim of this study.

## 2. Methods

Written informed consent was obtained from all persons before entering the study. The study was approved by the local ethics committee (Bolzano, number 19-2014) and performed in accordance with the Declaration of Helsinki.

### 2.1. Patients' Inclusion

All consecutive patients presenting to the Neurology Outpatient Clinic of Merano Hospital between December 2014 and November 2015 with a clinical diagnosis of optic neuritis were enrolled. Inclusion criteria were those adopted in the Optic Neuritis Treatment Trial by the Opticus Neuritis Study Group: 18–46 years of age; acute unilateral optic neuritis with visual symptoms for 8 days or fewer; a relative afferent pupillary defect and a visual field defect in the affected eye; no previous episodes of optic neuritis in the affected eye; no previous corticosteroid treatment for optic neuritis or multiple sclerosis; and no systemic disease other than multiple sclerosis that might be the cause of the optic neuritis [3].

All patients underwent neurologic and ophthalmologic examinations, including visual acuity assessment and direct ophthalmoscopy. Laboratory examinations included vasculitis screening, anti-neuromyelitis optical antibodies (aquaporin4-antibodies), and antibodies against myelin oligodendrocyte glycoprotein. MRI was done to exclude other causes of optic neuritis or compressive lesions [3]. All patients were followed up for 12 months.

### 2.2. Procedure of the Study and B-Ultrasound Sonography

Transorbital sonography, visual acuity, and biomarkers were assessed at onset (T0) and after 12 months (T12). At first presentation, transorbital sonography was evaluated by an expert neurosonologist (PL), who was accredited by the Italian Medical Ultrasound Society. We did not evaluate the sensitivity of sonographic data, but high attention was paid in order to assure quality control for the sonographic data. The high reliability of this technique has been reported in previous studies [15, 16]. In order to reduce the variability of measurements, the ONSD was measured three times in each eye and the mean values were calculated.

Transorbital sonography was always performed prior to the initiation of steroid treatment. Afterwards, all included patients received a single course of one gram of intravenous methylprednisolone sodium succinate over five days. The sonographer was unaware of the condition of case or control and of the affected side. To ensure blinding, patients and healthy controls were asked not to reveal their status (or their affected side) during examinations and were always placed on the examination table before the sonographer's arrival.

Transorbital sonography was carried out in B-mode using a Toshiba Applio XG equipped by a 4-11 Megahertz 5 S1 Linear Probe (Toshiba Medical System, Applio Nasu, Japan). We adopted the same procedure described elsewhere [6, 7].

### 2.3. Assessment of OPN and NfH

OPN and NfH levels were determined for all patients presenting with symptoms suggestive of acute unilateral optic neuritis and before the beginning of the infusion of methylprednisolone. After collection, the clotted venous blood was centrifuged at 3000 rpm for 15 min to obtain the serum that was then stored at −80°C until use. OPN and NfH serum levels were measured using the human OPN DuoSet ELISA development kit and the ELISA-pNFH-V1 (R&D Systems, Minneapolis, MN, USA; EnCor Biotechnology Inc., Gainesville, FL, USA, resp.) according to the manufacturer's instructions. The optical density (OD) of each sample was determined at 450 nm with a Spectra Count (Bio-Rad, Hercules, CA, USA), subtracting the lowest mean OD of the negative control. Sensitivity for OPN ELISA was 0.0625 ng/ml and for NfH ELISA was 0.2 ng/ml. Specificity of OPN ELISA kit was tested with recombinant human MMP-3 prepared at 50 ng/ml that showed no cross-reactivity or interference. Specificity of NfH ELISA kit was tested with HPLC-purified bovine NfM (7.8%), with bovine NfL (6.5%), and with bovine GFAP (0.06%) [17]. All samples were stored at the Clinic of Merano Hospital and then sent to the University of Novara. Due to a shortage of patients' samples, we decided to test OPN in all samples and NfH in 19 patients.

### 2.4. Statistical Evaluation

Continuous variables were described using median and interquartile range (IQR) for nonparametrically distributed variables and mean with standard deviation for parametrically distributed variables.

Comparisons between groups were assessed using the nonparametric Mann–Whitney *U* test and Wilcoxon signed-rank test. Correlations were assessed with the Spearman test; the level of statistical significance was set at *p* = 0.05.

All analyses were performed using dedicated statistical software (IBM Statistical Package for Social Science (SPSS), version 23.0.0.2, Armonk, New York, USA).

## 3. Results

### 3.1. Participants

Of the 28 patients who presented with a newly diagnosed optic neuritis during the study period, 5 did not meet the study criteria and were excluded from further analysis (1 missed during follow-up, 4 had potentially confounding conditions: 1 pituitary adenoma, 1 meningeal carcinomatosis, 1 neurosarcoidosis, and 1 tuberculum sellae meningioma). Hence, we had data from 23 patients, of whom 11 had prior MS relapses without previous optic neuritis and 12 had isolated optic neuritis. We analyzed data from 19 age-matched healthy controls. Demographic and clinical characteristics of the included patients and controls are reported in Table 1.

### 3.2. Ultrasound and Visual Acuity

Table 2 reports findings on ONSD, OND, and visual outcomes of both patients and controls. We observed a significant thickening of ONSD in the affected side (median 6.3 mm, IQR 6.0–6.5) compared to healthy controls (median 5.2 mm, IQR 4.8–5.5; *p* < 0.001) and after 12 months (median 5.3 mm, IQR 4.9–5.6; *p* < 0.001). The median OND in the affected side (3.4 mm, IQR 2.9–3.8) was significantly increased compared to the controls (median 2.7 mm, IQR 2.5–2.9; *p* < 0.001) and after 12 months of follow-up (median 2.8 mm, IQR 2.7–3.0; *p* < 0.01). Visual acuity in the affected eye was significantly worse at presentation (median 0.80, IQR 0.40–1.00) than after 12 months (median 1.00, IQR 0.90–1.00; *p* < 0.001).

### 3.3. Biomarkers

Serum OPN levels were significantly higher in patients with optic neuritis at presentation (median 6.44 ng/ml, IQR 2.05–10.06) compared to healthy controls (median 3.21 ng/ml, IQR 1.34–4.34; *p* < 0.03) (Table 3). Concentrations of NfH were significantly higher in patients (median 0.39 ng/ml, IQR 0.21–0.55) than in controls (median 0.02 ng/ml, IQR 0.02–0.08; *p* < 0.001) (Table 3). OPN and NfH concentrations remained elevated after 12 months with no statistically significant difference in OPN (*p* = 0.24) or NfH (*p* = 0.94) between T0 and T12.

### 3.4. Correlations between OPN, NfH, and ONSD

We found a statistically significant correlation between the ONSD value in the affected side and serum levels of OPN at T0 (*r* = 0.68; *p* < 0.05). Conversely, no statistically significant correlation between ONSD in the affected side and NfH level was observed (*r* = −0.43; *p* = 0.09).

### 3.5. OPN, NfH and Visual Acuity

Statistically significant differences were found in serum levels of OPN at T0 between patients with residual visual deficit and patients showing complete recovery (medians 7.7 versus 2.6 ng/ml; *p* = 0.03).

Regarding the serum levels of NfH at T0, we observed no statistically significant difference between patients with visual deficit at follow-up compared with patients with a good visual outcome (medians 0.39 versus 0.28 ng/ml; *p* = 0.291).

OPN values at T12 were significantly higher in patients with worsened visual acuity compared to patients with complete recovery of visual function (medians 10.8 versus 4.6 ng/ml; *p* = 0.019).

Regarding the serum levels of NfH at T12, we observed no statistically significant difference between the 7 patients with worsened visual acuity and patients who had regained normal visual acuity (medians 0.36 versus 0.25 ng/ml; *p* = 0.236).

## 4. Discussion

This is the first longitudinal study assessing circulating biomarkers of inflammation and axonal injury in parallel with transorbital sonography and visual acuity in patients with acute optic neuritis. Our approach is quite novel since we explored translational aspects that are well established in other neuroinflammatory conditions such as MS [18] but definitely less studied in optic neuritis [19].

Our study confirms that transorbital sonography is a sensitive technique for visualizing optic nerve thickening in the affected eye [7]. Nerve swelling is indeed the direct expression of the hallmark of optic neuritis, that is, inflammation. Furthermore, transorbital sonography can provide quantitative observations of the increase of nerve sheaths determined by the perineural subarachnoid fluid.

NfH and OPN levels were higher in patients with optic neuritis than in controls and remained elevated even after 12 months. Moreover, OPN concentration was significantly higher in patients with residual visual deficit after 12 months, thus supporting its detrimental effect in neuroinflammatory conditions [20]. Conversely, we did not find a higher concentration of NfH in patients with residual deficit compared to patients with a complete recovery after 12 months.

Our findings are in line with a previous study showing higher OPN levels during MS relapses and a direct correlation between OPN levels and disability [21]. Two further studies confirmed that OPN levels were higher in MS relapse compared to remission [13, 22].

Other studies found higher concentrations of NfH in patients with optic neuritis compared to controls or higher concentrations in patients with optic neuritis and neuromyelitis optica with more serious visual impairment compared to MS patients with optic neuritis or controls [2, 23]. Our study failed to show a significant relation between higher NfH concentrations at symptom onset and residual visual impairment. Conversely, we found a direct correlation between OPN and both ONSD and OND. Such parameters can provide complementary information on the dynamics of inflammation, and therefore, we suggest that they should be further studied in optic neuritis.

Notwithstanding the significant improvement in visual acuity, the persistence of high biomarkers concentrations over time suggests the persistence of underlying inflammation and neurodegeneration. The ONTT trial has indeed shown a spontaneous recovery in many but not all patients over a few weeks in acute idiopathic optic neuritis, even though the effect of treatment in subgroups was not analyzed [3].

The main limitation of this study is the small sample size. Therefore, our results need to be cautiously interpreted and replicated on a larger population. The use of transorbital sonography together with biomarkers would be of particular interest to monitor subgroups of patients with visual loss progressing for more than 2 weeks and with the absence of recovery for more than 3 weeks. Our findings were obtained by a single operator, but they can be easily replicated thanks to the low inter-rater variability of this technique [6].

Overall, our results support the usefulness of examining biomarkers in optic neuritis, since they provide complementary information on pathophysiology. The increase of OPN and ONSD seems to be correlated with the intensity of inflammation and might have a prognostic value, in analogy to what was suggested in MS [24]. Moreover, a similar study design could be easily applied to test correlations between ultrasound findings and other circulating molecules with a potential role in optic neuritis [25].

In conclusion, this study suggests that OPN levels predict visual outcome and neuronal loss after optic neuritis, whereas the role of NfH remains to be fully elucidated.

## Figures and Tables

**Table 1:** Characteristics of patients and controls.

	Optic neuritis	Healthy controls	*p* value
Number of patients, *n* (%)	23 (100)	19 (100)	
Sex			
Females, *n* (%)	19 (83)	13 (68)	0.47^∗^
Males, *n* (%)	4 (17)	6 (32)	
Time from onset days	14 (8–26)	—	
Follow-up days	360	—	
Age, years (mean ± SD)	33.0 ± 8.0	34.3 ± 9.2	0.63^∗∗^
Isolated optic neuritis, *n* (%)	12 (52.2)		
MS-optic neuritis, *n* (%)	11 (47.8)		

Data were expressed in frequency (percentage) or means ± standard deviation. ^∗^Fisher's exact test. ^∗∗^*t*-test for independent groups.

**Table 2:** Sonographic features and visual outcomes of patients and OPN and NfH values assessed at baseline (T0) and after 1 year (T12) of follow-up.

Parameter	T	ON patients affected eye (*n* = 23)	Healthy controls (*n* = 19)	*p* value
ONSD, mm	T0	6.3 (6.0–6.5)	5.2 (4.8–5.5)	<0.001
	T12	5.3 (4.9–5.6)		
	*p* value	<0.001		
OND, mm	T0	3.4 (2.9–3.8)	2.7 (2.5–2.9)	<0.001
	T12	2.8 (2.7–3.0)		
	*p* value	0.001		
Visual acuity	T0	0.80 (0.40–1.00)		
	T12	1.00 (0.90–1.00)		
	*p* value	<0.001		

T: time; ON: optic neuritis; ONSD: optic nerve sheath diameter, mm; OND: optic nerve diameter, mm; mm: millimeter; OPN: osteopontin; NfH: neurofilament heavy chain; ng/ml: nanogramm/milliliter; *n*: number of patients. Data are reported as median (IQR).

**Table 3:** OPN and NfH values assessed at baseline (T0) and after 1 year (T12) of follow-up.

Parameter	T	Patients^∗^	Healthy controls (*n* = 19)	*p* value
OPN, ng/ml	T0	6.44 (2.05–10.06)	3.21 (1.34–4.34)	<0.03
	T12	6.70 (3.85–10.90)		
	*p*	0.24		
NfH, ng/ml	T0	0.39 (0.21–0.55)	0.02 (0.02–0.08)	<0.001
	T12	0.3 (0.21–0.48)		
	*p*	0.94		

OPN: osteopontin; NfH: neurofilament heavy chain; ng/ml: nanogramm/milliliter; *n*: number of patients. Data are reported as median (IQR). ^∗^23 patients underwent OPN measurements at T0 and T12, and 19 patients underwent NfH measurements at T0 and T12.

## References

[1] Toosy A. T., Mason D. F., Miller D. H. (2014). Optic neuritis. *Lancet Neurology*.

[2] Petzold A., Rejdak K., Plant G. T. (2004). Axonal degeneration and inflammation in acute optic neuritis. *Journal of Neurology, Neurosurgery, and Psychiatry*.

[3] Optic Neuritis Study Group (2008). Multiple sclerosis risk after optic neuritis: final optic neuritis treatment trial follow-up. *Archives of Neurology*.

[4] Petzold A., Wattjes M. P., Costello F. (2014). The investigation of acute optic neuritis: a review and proposed protocol. *Nature Reviews Neurology*.

[5] Hickman S. J., Toosy A. T., Jones S. J. (2004). Serial magnetization transfer imaging in acute optic neuritis. *Brain*.

[6] Lochner P., Coppo L., Cantello R. (2014). Intra- and interobserver reliability of transorbital sonographic assessment of the optic nerve sheath diameter and optic nerve diameter in healthy adults. *Journal of Ultrasound*.

[7] Lochner P., Cantello R., Brigo F. (2014). Transorbital sonography in acute optic neuritis: a case-control study. *AJNR - American Journal of Neuroradiology*.

[8] Lochner P., Leone M. A., Coppo L. (2016). B-mode transorbital ultrasononography for the diagnosis of acute optic neuritis. A systematic review. *Clinical Neurophysiology*.

[9] Youl B. D., Turano G., Miller D. H. (1991). The pathophysiology of acute optic neuritis. An association of gadolinium leakage with clinical and electrophysiological deficits. *Brain*.

[10] Clemente N., Raineri D., Cappellano G. (2016). Osteopontin bridging innate and adaptive immunity in autoimmune diseases. *Journal of Immunology Research*.

[11] Boggio E., Dianzani C., Gigliotti C. L. (2016). Thrombin cleavage of osteopontin modulates its activities in human cells in vitro and mouse experimental autoimmune encephalomyelitis in vivo. *Journal of Immunology Research*.

[12] Modvig S., Degn M., Horwitz H. (2013). Relationship between cerebrospinal fluid biomarkers for inflammation, demyelination and neurodegeneration in acute optic neuritis. *PLoS One*.

[13] Vogt M. H., Floris S., Killestein J. (2004). Osteopontin levels and increased disease activity in relapsing-remitting multiple sclerosis patients. *Journal of Neuroimmunology*.

[14] Petzold A., Plant G. T. (2012). The diagnostic and prognostic value of neurofilament heavy chain levels in immune-mediated optic neuropathies. *Multiple Sclerosis International*.

[15] Bäuerle J., Lochner P., Kaps M., Nedelmann M. (2012). Intra- and interobsever reliability of sonographic assessment of the optic nerve sheath diameter in healthy adults. *Journal of Neuroimaging*.

[16] Bäuerle J., Schuchardt F., Schroeder L., Egger K., Weigel M., Harloff A. (2013). Reproducibility and accuracy of optic nerve sheath diameter assessment using ultrasound compared to magnetic resonance imaging. *BMC Neurology*.

[17] Petzold A., Keir G., Green A. J., Giovannoni G., Thompson E. J. (2003). A specific ELISA for measuring neurofilament heavy chain phosphoforms. *Journal of Immunological Methods*.

[18] Castelli L., Comi C., Chiocchetti A. (2007). ICOS gene haplotypes correlate with IL10 secretion and multiple sclerosis evolution. *Journal of Neuroimmunology*.

[19] Rossi S., Motta C., Studer V. (2014). Interleukin-8 is associated with acute and persistent dysfunction after optic neuritis. *Multiple Sclerosis*.

[20] Carecchio M., Comi C. (2011). The role of osteopontin in neurodegenerative diseases. *Journal of Alzheimer’s Disease*.

[21] Shimizu Y., Ota K., Ikeguchi R., Kubo S., Kabasawa C., Uchiyama S. (2013). Plasma osteopontin levels are associated with disease activity in the patients with multiple sclerosis and neuromyelitis optica. *Journal of Neuroimmunology*.

[22] Vogt M. H., Lopatinskaya L., Smits M., Polman C. H., Nagelkerken L. (2003). Elevated osteopontin levels in active relapsing-remitting multiple sclerosis. *Annals of Neurology*.

[23] Pasol J., Feuer W., Yang C., Shaw G., Kardon R., Guy J. (2010). Phosphorylated neurofilament heavy chain correlations to visual function, optical coherence tomography, and treatment. *Multiple Sclerosis International*.

[24] Comi C., Cappellano G., Chiocchetti A. (2012). The impact of osteopontin gene variations on multiple sclerosis development and progression. *Clinical and Developmental Immunology*.

[25] Bellavista E., Santoro A., Galimberti D., Comi C., Luciani F., Mishto M. (2014). Current understanding on the role of standard and immunoproteasomes in inflammatory/immunological pathways of multiple sclerosis. *Autoimmune Diseases*.

